# Hospital-acquired SARS-CoV-2 pneumonia in a person living with HIV

**DOI:** 10.1177/0956462420957528

**Published:** 2020-09-21

**Authors:** Alfredo Juan Chiappe Gonzalez, Juan Jose Montenegro-Idrogo, Ademir Roberto Vargas Vadillo, Milagros Slee Torres, Ivan Vargas Matos, Cristhian Pedro Resurrección Delgado

**Affiliations:** 1 Hospital Nacional Dos de Mayo, Lima, Peru; 2 Clínica Angloamericana, Lima, Peru

**Keywords:** Combination ART (cART), HIV (Human immunodeficiency virus), Location, Other, South America, Viral disease

## Abstract

We report the case of a 38 year-old man living with well-controlled HIV on antiretroviral therapy who developed cerebellar symptoms and was admitted to hospital for management of an intraventricular cryptococcoma. During his hospital stay he contracted SARS CoV-2 infection within the hospital setting with a fatal outcome.

## Introduction

SARS-CoV-2 is a novel viral infection responsible for the current COVID-19 pandemic. In Latin America, the first confirmed case was reported on February 25, 2020 in a Brazilian man who traveled to Lombardy, northern Italy.^1^ In Peru, the first confirmed case occurred on March 6, 2020. The following day a state of national emergency was declared, followed by a national quarantine implemented on March 16.^2^ Medical personnel and patient visits by family members are an important at-risk population at risk of COVID-19 due to close contact with patients in daily practice.^3^ The aforementioned is the reason why hospital-acquired infection is possible, as are hospital outbreaks. Outbreaks of this disease within hospitals are of high epidemiological relevance due to the abrupt cessation risk of local medical care, both at the hospital level, and progressively to its extent and repercussions at a higher social level.^3^,^4^ We report a case of SARS-CoV-2 hospital-acquired pneumonia in a person living with HIV, admitted to a Peruvian public hospital on February 13, almost one month prior to the pandemic's arrival to our country.

## Case report

A 38-year-old male from Lima, Peru living, with HIV on antiretroviral therapy (ART) (tenofovir-DF/emtricitabine + atazanavir/ritonavir) for several years, was admitted to our hospital on February 13, 2020 after presenting with a cerebellar syndrome characterized by ataxia, dysmetria and headache of moderate intensity. The updated immunologic and virologic studies revealed a CD4 count of 438 cells/μL and an undetectable HIV viral load, respectively. No respiratory signs or symptoms were present on admission.

Brain magnetic resonance (MRI) was performed, showing significant obstructive hydrocephalus with a nonspecific nodular hypo intensity with no gadolinium contrast enhancement in the 4th ventricle. In addition, cerebrospinal fluid was positive for *Cryptococcus neoformans* in the lateral flow study. Therefore, a diagnosis of intraventricular cryptococcoma was established, and the patient initiated antifungal therapy with amphotericin B deoxycholate plus fluconazole, while awaiting a ventriculoperitoneal shunt procedure. The patient remain hospitalized during this waiting period.

**Figure 1.**
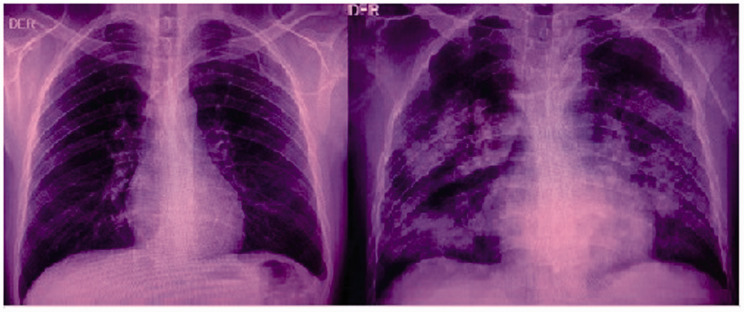
Chest radiographs. Baseline (left) and day 8 images (right).

**Figure 2.**
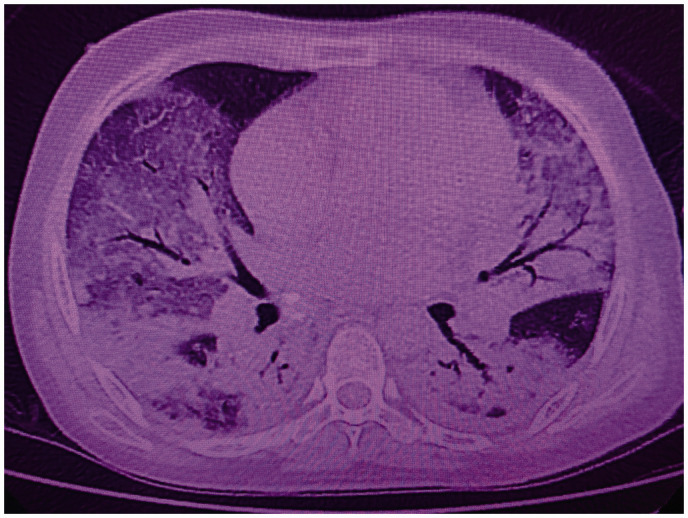
Chest computed tomography-scan at the end of the second week of disease.

On March 22th, without having received recent visits from relatives, with the patient developed malaise and generalized weakness, sore throat and nonproductive cough that appeared on day 3 and 4, respectively. On the 7th day, the patient developed fever and, consecutively, on the 8th day, dyspnea and diarrhea. No nasal secretion was seen. Laboratory findings were relevant for lymphopenia, thrombocytopenia and increased levels of C-reactive protein, urea, fibrinogen, lactate dehydrogenase and D-dimer. Normal values of leukocytes, aspartate aminotransferase, alanine aminotransferase and creatinine were reported. Chest radiograph and computed tomography scan of the chest show bilateral ground-glass opacities with bilateral patchy alveolar infiltrates (Figures 1 and 2). SARS-CoV2 pneumonia was suspected at this point. Non-invasive oxygen supplementation was required, and symptomatic therapy was provided to the patient. Finally, positive results from RT-PCR SARS-CoV-2 tests from the throat and nasal swabs were obtained on March 31, confirming the diagnosis of SARS-CoV-2 pneumonia.

During follow up, the patient's pulmonary compromise worsened progressively, and he ultimately passed away on April 4, 2020 despite the use of high dose corticosteroids, not being able to find a mechanical ventilation device available at the institution.

## Discussion

Little is known about HIV and SARS-CoV-2 coinfection. The Centers for Disease Control and Prevention (CDC) has highlighted the risk of people living with HIV (PLHIV) regarding the infection and development of complications during the course of SARS-CoV-2 disease.^5^ ART has extended the life expectancy of these patients, which has been accompanied by an increase in the prevalence of chronic illness such as diabetes mellitus, high blood pressure, pulmonary and heart diseases. These comorbidities have been associated with an increased risk of an unfavorable course of COVID-19 disease.^5^,^6^ Blanco et al. reported different complications in PLHIV with adequate virological control, as in the case presented, with a protease inhibitor as the HIV treatment core regimen.^6^ Likewise, a randomized controlled trial (RCT) pointed out the ineffectiveness of lopinavir/ritonavir in improving mortality at 28 days in the treatment of severe COVID-19 pneumonia,^7^ which results a great challenge in choosing the best therapeutic alternative in this viral coinfection scenario.

Hospital-acquired SARS-CoV-2 pneumonia has not been specifically described. There are several strategies to protect hospitals from COVID-19 outbreaks. The reinforcement in the detection of other respiratory viruses and the improvement in the care of these patients through the optimization of biosecurity measures of health workers are some examples.^8^ The Chinese experience illustrates preventive recommendations for intrahospital transmission among health workers and non-COVID patients through various control measures. Examples of these are reorganization of the movement of patients and hospital personnel, establishment of a specific geolocalization for patients with respiratory symptoms, the appropriate implementation of personal protective equipment according to exposure, training of health workers in measures to prevent infection, disinfection of different hospital areas, the limitation of visits by relatives of patients, among others.^9^

Hospitals generally employ multiple approaches to prevent health care-associated infections. However, high patient volume could result in unsuccessful control.^3^,^4^ Therefore, it is extremely important to document cases of in-hospital transmission of SARS CoV-2, in order to implement additional actions that favor the optimization of the prevention of viral transmission between COVID and non-COVID patients within the hospital environment. Our case demonstrates the morbidity and mortality of SARS CoV-2 in a patient who contracted SARS CoV-2 within the hospital setting.
